# Supplementary material for: Osteosarcoma Cells and Undifferentiated Human Mesenchymal Stromal Cells Are More Susceptible to Ferroptosis than Differentiated Human Mesenchymal Stromal Cells
Source: Antioxidants (Basel). 2025 Feb 6;14(2):189. doi: 10.3390/antiox14020189 (PMC11852062; doi:10.3390/antiox14020189)
Supplement: Supplementary file 1 [file antioxidants-14-00189-s001.zip › antioxidants-3318322-supplementary.pdf]

Supplementary materials

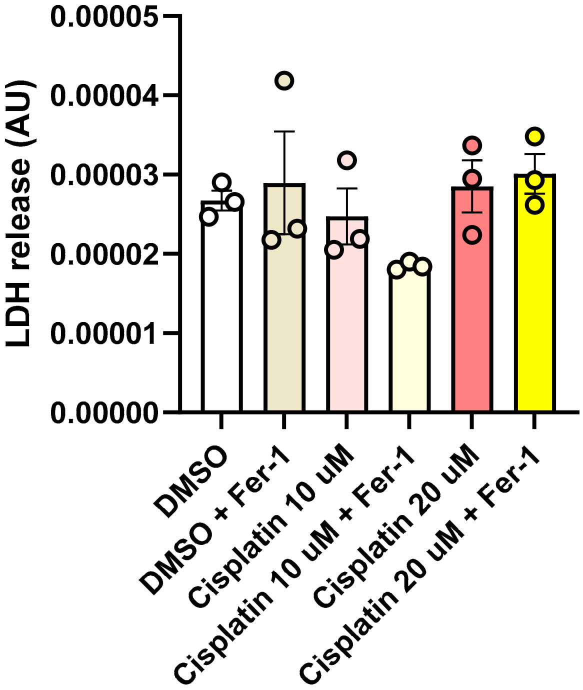

Figure S1. Effect of cisplatin, a GPX4 inhibitor, on the LDH release in the MG63 cells. The levels of LDH release, remained at the control levels. Addition of ferrostatin-1 did not change LDH levels as well. Data are presented as means ± SEM (error bars). Statistical evaluation was performed with one way ANOVA followed by post hoc Holm–Sidak's multiple comparisons test. We did not find any significant difference.

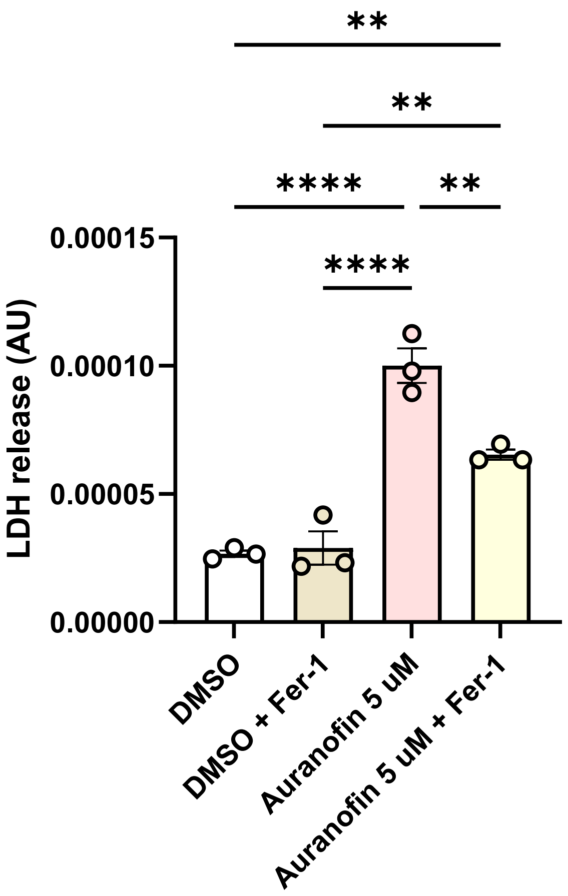

Figure S2. Effect of Auranofin, a GSTP1 inhibitor, on the LDH release in the MG63 cells. Substantial increase in LDH release reflects elevated levels of cell death. The release of LDH is partially reversible upon treatment with ferrostatin-1. Data are presented as means ± SEM (error bars). Statistical evaluation was performed with one way ANOVA followed by post hoc Holm–Sidak's multiple comparisons test. **—p ≤ 0.01; ****—p ≤ 0.0001.

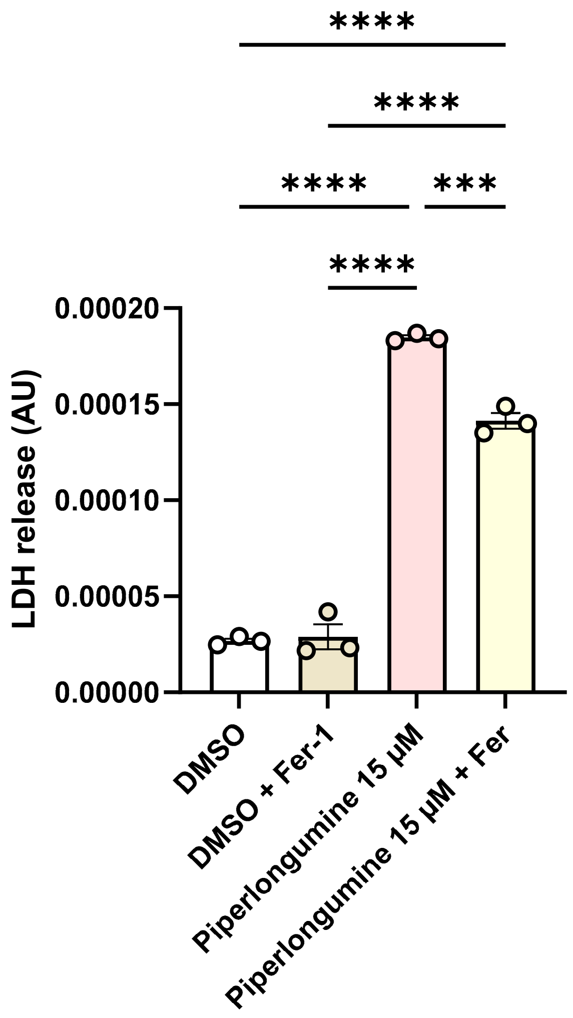

Figure S3. Effect of Piperlongumine, a GSTP1 inhibitor, on the LDH release in the MG63 cells. Substantial increase in LDH release reflects elevated levels of cell death. The release of LDH is partially reversible upon treatment with ferrostatin-1. Data are presented as means ± SEM (error bars). Statistical evaluation was performed with one way ANOVA followed by post hoc Holm–Sidak's multiple comparisons test. ***—p ≤ 0.001; ****—p ≤ 0.0001.
